# Models of gouty nephropathy: exploring disease mechanisms and identifying potential therapeutic targets

**DOI:** 10.3389/fmed.2024.1305431

**Published:** 2024-02-29

**Authors:** Lin Wang, Xiaoyu Zhang, Jiayan Shen, Yuanyuan Wei, Ting Zhao, Niqin Xiao, Xiaoman Lv, Dongdong Qin, Yundong Xu, Yang Zhou, Jing Xie, Zhaofu Li, Zhaohu Xie

**Affiliations:** Yunnan University of Chinese Medicine, Kunming, Yunnan, China

**Keywords:** gouty nephropathy, pathogenesis, animal models, cell model, uric acid

## Abstract

Gouty nephropathy (GN) is a metabolic disease with persistently elevated blood uric acid levels. The main manifestations of GN are crystalline kidney stones, chronic interstitial nephritis, and renal fibrosis. Understanding the mechanism of the occurrence and development of GN is crucial to the development of new drugs for prevention and treatment of GN. Currently, most studies exploring the pathogenesis of GN are primarily based on animal and cell models. Numerous studies have shown that inflammation, oxidative stress, and programmed cell death mediated by uric acid and sodium urate are involved in the pathogenesis of GN. In this article, we first review the mechanisms underlying the abnormal intrinsic immune activation and programmed cell death in GN and then describe the characteristics and methods used to develop animal and cell models of GN caused by elevated uric acid and deposited sodium urate crystals. Finally, we propose potential animal models for GN caused by abnormally high uric acid levels, thereby provide a reference for further investigating the methods and mechanisms of GN and developing better prevention and treatment strategies.

## 1 Introduction

Gouty nephropathy (GN), also known as uric acid nephropathy, encompasses a range of renal disorders caused by abnormal purine metabolism. This condition results from either excessive uric acid production or decreased excretion, leading to the deposition of urate crystals within the lumen of distal tubules or collecting ducts (1). In the early stages of GN development, interstitial damage is the mainstay. However, as the disease progresses, it can also affect renal glomerulus, glomerular mesangial cell proliferation leads to the widening of the mesangial area, accompanied by neutrophil infiltration. The proliferative mesangial tissue gradually extends to the surrounding capillaries, invading the space between the capillary basement membrane and endothelial cells. The proliferative mesangial tissue can envelop all the capillary walls, resulting in the thickening of the capillary basement membrane in the glomeruli and diffuse proliferation of mesangial and endothelial cells. This progression leads to persistent proteinuria and may culminate in renal failure (2). Persistently high blood uric acid levels serve as the pathological basis not only for the development of gout but also for other related disorders. Renal damage has been reported in approximately 86.3% of gout patients (3). The prevalence of GN is notably higher in oceanic island countries compared to developing countries, with a particularly significant burden among indigenous populations, such as Taiwan’s indigenous people and Maori people, where prevalence exceeds 10% (4). GN poses a serious threat to human health. Therefore, GN warrants focused attention and extensive research.

Currently, the prevailing view is that the onset and progression of GN are closely related to the abnormal activation of the intrinsic immune system (5). This activation can induce an inflammatory response in the presence of excessively high blood uric acid levels and/or local deposition of monosodium urate (MSU), resulting in varying degrees of damage to glomerular, tubular, and endothelial cells (6). These cascading events ultimately lead to GN. However, the precise pathogenesis of this condition has not been elucidated. Consequently, the primary approach to treatment revolves around symptom management, including uric acid reduction, urine alkalization, circulation improvement, and anti-inflammatory treatments (7). Unfortunately, there is a lack of specific drugs for GN. Hence, further exploration of the pathogenesis of this disease holds significant promise for the development of efficient new drugs and advanced strategies for prevention and treatment. Drawing upon research experiences from both domestic and international related disease models, we explore the use and modeling techniques of animal and *in vitro* cultured cell models used in GN-related research. Our objective is to identify a foundation for establishing GN models that conform to clinical characteristics, thereby offering valuable insights into methods and mechanisms for advancing GN research and the development of more effective prevention and treatment strategies.

## 2 Overview of the pathogenesis of GN

Uric acid is the final product of purine metabolism within the human body. Dysregulation in purine metabolism can lead to either increased or decreased serum uric acid excretion, ultimately resulting in hyperuricemia. In the human kidneys, approximately 90% of uric acid is reabsorbed into the bloodstream, with only 8–12% excreted by the kidneys (8). Clinical studies have shown that the increase in uric acid levels leads to the saturation of blood uric acid which results in the precipitation and deposition of urate crystals within renal tissue. This process leads to renal tubule injury, glomerulosclerosis, and renal interstitial fibrosis, culminating in gouty nephropathy (9). Long-term hyperuricemia induces blockage of renal tubules due to the deposition of urate crystals. Through ultrasound examinations, kidney stones have been detected in patients with gouty nephropathy biopsies. Staining kidney sections revealed tubular dilation, interstitial fibrosis, and inflammatory cell infiltration. In induced GN rats, urate deposition was mainly observed in renal tubules (10). Moreover, external factors such as prolonged consumption of excessive high-purine foods, obesity, and high fructose beverage intake have been implicated in clinical studies, and the use of drugs affecting acid metabolism, such as hydrochlorothiazide diuretics, salicylic acid, and lactic acid, can increase the burden on the kidneys. This increased burden can lead to a compensatory state, ultimately reducing renal filtration function and causing pathological changes. These urate crystals may be recognized by the immune system as dangerous signals, prompting the synthesis and release of inflammatory factors, thereby fostering inflammation (8). This, in turn, affects the cellular morphology and function of kidney tissue. The onset of GN involves multiple factors, with abnormal activation of the innate immune system and oxidative stress being the most important pathogenic factors. Programmed cell death is also closely related to the occurrence and progression of GN.

### 2.1 Abnormal activation of the innate immune system

The innate immune system constitutes a natural defense system mechanism developed by the body during evolution. It serves as a first line of defense against external pathogens and hosts tissue damage while also participating in the removal of damaged or aging cells within the body and regulating specific immune responses. Dysregulation of the innate immune system can trigger a pro-inflammatory response, leading to autoimmune diseases (11). Activation of the innate immune system primarily relies on pathogen-associated molecular patterns (PAMP) and damage-associated molecular patterns (DAMP). When cells are damaged or die, the molecular structure within these cells changes, releasing proteins, nucleic acids, and other molecules outside the cells. These molecules, recognized by the immune system as PAMPs and DAMPs, activate the immune system by binding to specific receptors on the surface of immune cells, thus triggering inflammatory reactions. Sodium urate, a metabolic product derived from nucleic acid degradation, is recognized by NLRP3 receptors (NOD-,LRR-and pyrin-domain-containing protein 3) on immune cells, activating the innate immune system. Evidence from animal studies suggest that urate crystals deposited in renal tubules and interstitial regions induce macrophage and T cell aggregation and activation, resulting in the recruitment of T cells and the production of chemokines. CCL chemokines play a pivotal role in the regulation of renal injury, participating in the activation of infiltrating cells associated with immune responses during inflammatory kidney injury (12). The activation of NLRP3 inflammasomes and the release of IL-1β during the inflammatory response process are crucial in the occurrence and development of GN. The first signal in this activation process seems to rely on the production of mitochondrial reactive oxygen species (ROS) and the upregulation of NLRP3 expression through signaling pathways involving Toll-like receptors (TLR) and myeloid differentiation response (MyD88) nuclear factors κB (NF-κB) (13). The second signal involves the activation of the NLRP3 inflammasome via pattern recognition receptors (PRR) recognizing a multiprotein complex comprising pathogen-associated molecular pattern (PAMP) and damage-associated molecular pattern (DAMP). This complex includes the sensors NLRP3, adapter apoptosis-related mirror protein (ASC), and cysteine asparagine-1. Activated cysteine asparagine-1 mediates the maturation and secretion of the pro-inflammatory cytokines IL-1β and IL-18 (14). In mice models, NLRP3 inflammasome activators trigger the production of mitochondrial ROS in cells, with the primary source of ROS being mitochondrial oxidative phosphorylation during electron transfer. External stimuli can lead to mitochondrial damage and release of excessive ROS, triggering inflammasome activation (15), further contributing to kidney damage.

**FIGURE 1:**
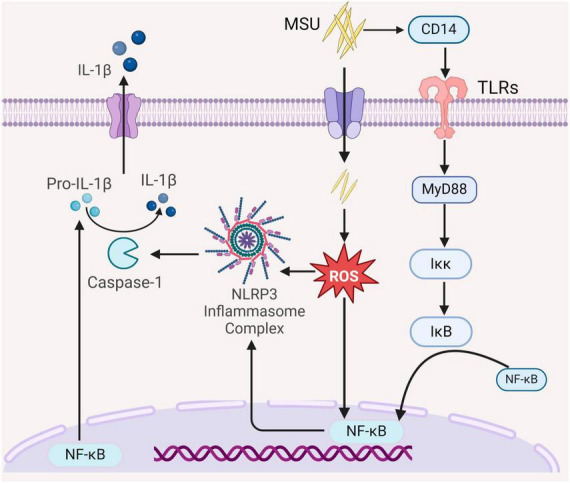
The role of the intrinsic immune system in the pathogenesis of GN. MSU activates the NF-κB signaling pathway by binding CD14 to Toll-like receptors (TLRs) on the cell membrane to recruit bone marrow differentiation response (MyD88), further activation Iκκ, and activated Iκκ cause phosphorylation of IκB to release NF-κB into the nucleus, which regulates the NF-κB signaling pathway, which modulates nuclear transcription and synthesis of interleukin-1β (Pro-IL-1β). Elevated uric acid levels induce oxidative stress and activate the NF-κB signaling pathway, thereby enhancing NF-κB nuclear transcription and activating the NLRP3 inflammasome, leading to the production of caspase-1. This, in turn, promotes the maturation and release of IL-1β, thereby exacerbating the inflammatory response (All figures were created using the BioRender).

Chronic high levels of uric acid in the peripheral circulation induce oxidative stress, a recognized risk factor for renal injury (16). *In vitro* experiments have demonstrated that uric acid can cause vascular endothelial cell injury by activating the NLRP3/IL-1β signaling pathway or Nicotinamide adenine dinucleotide phosphate (NADPH) enzymes (17). Concurrently, mitogen-activated protein kinase (MAPK) serves as a key regulatory pathway for cellular energy metabolism (16). A decrease in uric acid levels triggers the activation of AMPK (18). To comprehensively understand the disease’s developmental process and provide valuable insights for its prevention and treatment, it is essential to delve into renal inflammatory responses, oxidative stress, and related signaling pathways. Numerous animal experiments have demonstrated that mice with elevated uric acid concentrations exhibit significant renal dysfunction, cellular infiltration, and high expression of pro-inflammatory factors (19). Uric acid activates the NF-κB signaling pathway by stimulating NLRP3 inflammasome. Toll-like receptors (TLRs) structural domains are downstream pathways required for the activation of the NF-κB signaling pathway (20). MyD88 serves as a regulator of the NF-κB signaling pathway. In the presence of abnormal uric acid metabolism, TLRs activate Iκκ by recruiting MyD88, ultimately leading to the phosphorylation of I κB and the release of NF-κB into the nucleus (9). The activation of NF-κB results in the production of a large number of pro-inflammatory mediators, including cytokines, chemokines, and adhesion factors such as IL-1β, IL-6, IL-8, and monocyte chemoattractant protein-1 (MCP-1), etc. This surge in inflammatory cytokines, coupled with increased renal vascular permeability, elevates the risk of renal tubulointerstitial fibrosis. Animal studies have shown that elevated uric acid levels in mice lead to renal dysfunction. The accumulation of stones in renal tubules stimulates epithelial cells to produce MCP-1, contributing to renal injury alongside macrophage infiltration (Figure 1) (21).

### 2.2 Oxidative stress

Uric acid possesses both oxidative and antioxidant properties. It acts as a scavenger of oxygen radicals, protecting extracellular superoxide dismutase (SOD) function and structure, thereby preventing free reactive oxygen species from causing cellular damage. However, when uric acid concentrations exceed normal levels, it exhibits pro-oxidant effects, increasing the production of reactive oxygen species (ROS). Oxidative stress results from the overproduction of ROS or an increase in pro-oxidants, generating harmful free radicals that can damage cellular proteins, lipids, and DNA (22). Mitochondria are the primary source of reactive oxygen species within cells (23), with the main forms of ROS being hydroxyl radicals, hydrogen peroxide, and superoxide anion (24). The effects of oxidative stress are closely linked to inflammation and the risk of cardiovascular disease, conditions frequently associated with oxidative stress. The kidney, being a highly oxygen-consuming organ (25), plays a critical role in providing energy for a variety of cellular functions, including the removal of excess substances, reabsorption of nutrients, and the maintenance of fluid metabolism balance (26). Renal tubules, rich in mitochondria, can suffer tubular dysfunction induced by increased mitochondrial superoxide levels (27). Studies have demonstrated decreased mitochondrial oxygen consumption and ubiquitinase activity in renal cortical tissues in a rat model of hyperuricemia induced by uric acid (28). Jiang et al. (29) reported that mitochondrial dysfunction and organelle stress suppress the production of ATP levels and increase ROS levels, leading to renal insufficiency through *in vitro* experiments. Maintaining renal mitochondrial homeostasis is considered a therapeutic target for renal injury associated with oxidative stress (26).

The effects of soluble uric acid primarily manifest in vascular smooth muscle cells (VSMC), endothelial cells, adipocytes, and renal tubular epithelial cells (30). Uric acid-mediated oxidative stress activates the pro-inflammatory pathway of the uric acid transporter, damaging VSMCs and promoting cell proliferation. One study demonstrated that VSMC damage was associated with excessive oxygen free radicals and superoxide overproduction, accelerating nitric oxide degradation, thus reducing nitric oxide bioavailability. This oxidative stress was attributed to uric acid, mediating VSMC proliferation through the stimulation of renin-angiotensin (31). NADPH oxidase catalyzes superoxide and other reactive oxygen species, with NAPDH oxidase 4 (NOX4) being the most significant isoform in the kidney, located in renal tubules, renal fibroblasts, and glomerular thylakoid cells (32). Animal studies have demonstrated that excessive production of ROS and heightened inflammation are directly linked to NADPH, making renal oxidative stress a key contributor to renal injury and inflammation (Figure 1) (22).

### 2.3 Programmed cell death

#### 2.3.1 Apoptosis

**FIGURE 2:**
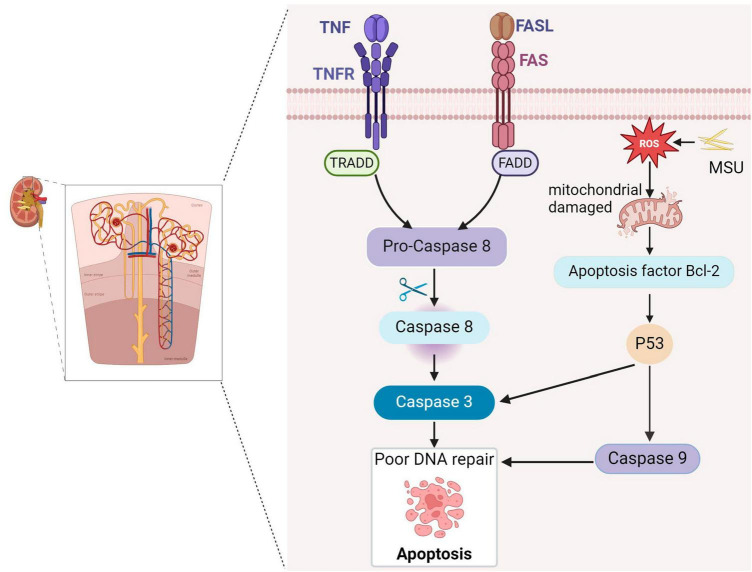
Gouty nephropathy (GN) cell apoptosis. Tumor necrosis factor receptor (TNFR) and factor-associated apoptosis (Fas) receptors induce apoptosis by binding to their respective ligands and cleaving Pro-Caspase-8 through the death domains TNF-associated death domain (TRADD) and Fas-associated death domain (FADD), producing active caspase-8 and activating caspase-3, triggering apoptosis. MSU causes oxidative stress and impairs mitochondrial function, leading to the release of the pro-apoptotic factor Bcl-2 (B-cell lymphoma-2), activating the P53 transcription factor, promoting caspase-9 and caspase-3, and ultimately triggering apoptosis.

Apoptosis is a critical process in which cells initiate their orderly death, regulated by specific genes. It serves to maintain the stability of the internal cellular environment and plays essential roles in individual growth, development, and aging within the immune system (33). For example, apoptosis can be induced in T and B cells to regulate immune responses (34). Apoptosis primarily involves two classical regulatory pathways: the endogenous mitochondrial pathway and the exogenous death receptor pathway (13). In the exogenous pathway, death receptors are transmembrane proteins that convey death signals via their cytoplasmic death domains. The tumor necrosis factor (TNF) receptor family, including TNF and Factor-related Apoptosis (Fas), is critical in this process. These ligands bind to membrane receptors and transmit death signals through proteins like TNF-related death structure domain (TRADD) and Fas-related death structure domain (FADD), and initiate the key component of apoptosis, cysteinase, to affect programmed cell death. TNF ligands bind to membrane receptors, and FADD recruits Pro-Caspase-8 downstream through the interaction of the death domain, which cleaves Pro-Caspase-8 to active Caspase-8, leading to cell death. In addition, the Fas ligand triggers cell death by binding to the Fas receptor (CD95) via a pathway similar to that of Fas (35). The endogenous pathway involves the migration of pro-apoptotic factors into the cytoplasm, stimulated by intracellular signals that disrupt mitochondrial membranes, allowing these factors to move into the cytoplasm (35). However, few studies have explored the role of apoptosis in gouty nephropathy. Animal experiments have revealed that oxidative stress resulting from elevated uric acid levels can affect mitochondrial function and structure, leading to apoptosis of renal tubular epithelial cells (28). Consequently, damage to tubular epithelial cell damage may be an essential link in the development of renal damage associated with hyperuricemia (HUA). Impaired tubular epithelial cells can activate the innate immunity through pattern recognition receptors, and stimulate the secretion of several pro-inflammatory and chemokines, as well as recruitment of inflammatory cells to infiltrate the interstitium. This promotes the pathogenesis of tubulointerstitial fibrosis (36). In addition, the epithelial-mesenchymal transition alters renal tubular epithelial cells and loss of epithelial characteristics which hinders the transformation into mesenchymal-like cells. Some studies have demonstrated that the expression of α smooth muscle actin is increased in renal tubular epithelial cells from the kidneys of rats with hyperuricemia. It is postulate that uric acid induces the transformation of epithelial cell phenotype, which may be one of the mechanisms leading to kidney damage. In animal experiments Increased mitochondrial P53 protein expression in rats fed with yeast and adenine, a source of uric acid, regulates the expression of the downstream proteins such as Caspase-3/-9, leading to the appearance of apoptotic vesicles in renal tubular epithelial cells and ultimately renal disease (37). MSU crystals can promote renal cell apoptosis by stimulating the mitochondrial cysteinase apoptotic pathway. Following MSU crystal stimulation, human embryonic kidney cells (HEK293) show increased expression of ROS, cysteinase-3, and cysteinase-9 expression (Figure 2) (38).

#### 2.3.2 Autophagy

**FIGURE 3:**
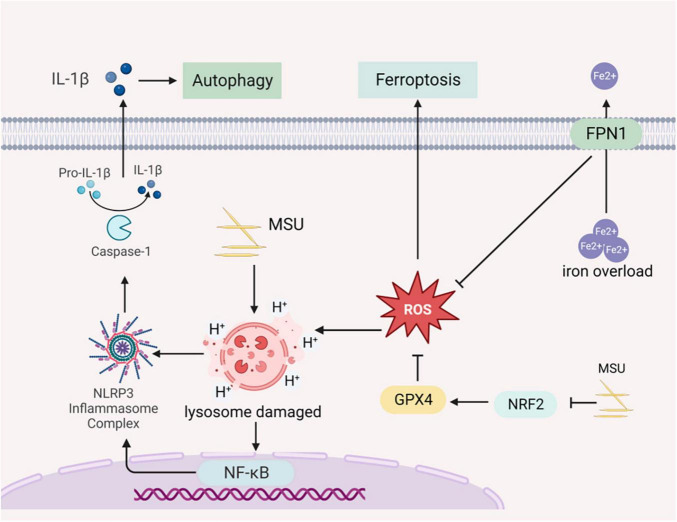
Gouty nephropathy (GN) cell autophagy and ferroptosis. Sodium urate prompts lysosomal swelling and rupture, releasing acidic contents into the cytoplasm. This triggers the activation of NLRP3 inflammasome, leading to the production and release of inflammatory cytokines into the extracellular space, causing cellular autophagy. FPN1 (Ferroportin 1), an iron-excretory protein, plays a crucial role in regulating iron transport. Dysfunctional FPN1 can result in the accumulation of intracellular Fe^2+^, leading to the accumulation of ROS and inducing ferroptosis. Sodium urate can induce ferroptosis by inhibiting the nuclear factor erythroid 2 related factor (NRF2) transcription factor and increasing lipid accumulation in cells.

Autophagy is a cellular process regulated by autophagy-related genes that involve the degradation of damaged organelles and macromolecules within lysosomes. It is a crucial pathway for maintaining cellular metabolic processes, intracellular physiological activity, and homeostasis. Autophagy is considered a cell survival mechanism with implications for both human health and disease (39). Under normal physiological conditions, autophagy degrades aging damaged organelles such as the endoplasmic reticulum, mitochondria, and peroxisomes, as well as soluble macromolecules including proteins and lipids. This degradation occurs within lysosomes and serves to maintain intracellular homeostasis (16, 17). In cases where the organism produces large amounts of uric acid due to increased purine catabolism, uric acid can lead to the formation of urate crystals in the kidneys. These monosodium urate crystals can rupture lysosomes, activating inflammasome and triggering the secretion of proinflammatory cytokines. Autophagy participates in the removal of damaged lysosomes, thereby suppressing inflammation and protecting the kidney in rats (40). Although autophagy is essential for normal development and survival, there is emerging evidence suggesting that it may also contribute to cell death during disease pathogenesis (41). When autophagy becomes over-activated, it can lead to excessive self-digestion of cellular material within lysosomes, causing cell swelling and rupture, and the breakdown of organelles and cellular structures beyond a sustainable point (42). Researchers have established criteria for autophagic cell death, which include not activating or relying on other programmed cell death pathways, stimulating cell death by increasing autophagic flux, and being capable of rescuing or preventing cell death by blocking autophagy through autophagy proteins (43), Autophagic cell death has been observed in lower organisms such as Drosophila midgut, where the inhibition of autophagy through the knockdown of the lipid transporter protein ATG2 delayed tissue lysis, highlighting the critical regulatory role of autophagy in cell death (44). A study of a rat model showed that TGF-β1 stimulated protein kinase Cα (PKCα) signaling and increased autophagic flux in the kidney mesenchymal fibroblasts NRK-49F, thereby promoting renal fibrosis (Figure 3) (45).

#### 2.3.3 Pyroptosis

**FIGURE 4:**
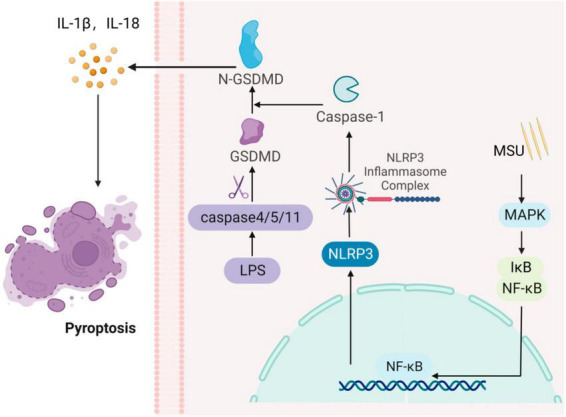
Gouty nephropathy (GN) cell pyroptosis. The formation of inflammasome activates Caspase-1, which cleaves the gasdermin D (GSDMD) protein, activating its N-terminal structural domain, GSDMD-N. GSDMD-N then forms pores in the cell membrane, leading to membrane rupture and the release of IL-1β and IL-18 pro-inflammatory cytokines, inducing cellular pyrolysis. Simultaneously, cytoplasmic lipopolysaccharide (LPS) activates Caspase-4/5/11, which cleaves the GSDMD protein, triggering cellular pyroptosis.

Pyroptosis is a primarily proinflammatory form of cell death mediated by the gasdermin D (GSDMD) protein, regulated by cysteine proteases (caspases) (46). It is associated with the release of inflammatory mediators and cell death, triggering an inflammatory response in the organism. Pyroptosis is a vital component of innate immunity, protecting the organism from pathogenic microorganisms (47). Pyroptosis is characterized by the formation of localized pores in cell membranes, cellular swelling, rupture, and the release of proinflammatory cytokines. When over-activated, it can lead to tissue damage and the development of chronic diseases (48). Initially, pyroptosis was thought to be a Caspase-1-mediated pathway of macrophage and monocyte death, and inflammatory vesicle-dependent cell death, characterized by a lack of cell membrane integrity and the secretion of inflammatory factors (IL-1β) (49). In response to stimulation by endogenous or exogenous danger signals, NLRP3 inflammasome can activate GSDMD proteins. The activated GSDMD protein binds to membrane lipids, cleaves liposomes, and forms pores in the membrane, leading to cell swelling and rupture (50). This process also causes the release of substances such as inflammatory factors into the extracellular space, recruiting inflammatory cells and amplifying the inflammatory response. Moreover, Caspase-11/4/5 can act as a sensor to detect cytoplasmic lipopolysaccharide (LPS) released by Gram-negative bacteria (48, 51). Activated Caspase-11/4/5 can cleave GSDMD proteins and activate NLRP3 inflammasome, leading to the release of inflammatory cytokines (IL-1β/-18) and cell death, independent of Caspase-1. Furthermore, elevated uric acid levels can activate NLRP3 inflammasome in endothelial cells, resulting in increased expression of associated inflammatory factors. This process can activate GSDMD proteins, ultimately inducing endothelial cell scorching by a mechanism that has been validated. *In vitro* AKI cell model showed that the Caspase-1/GSDMD signaling pathway participates in pyroptosis associated with uric acid nephropathy (Figure 4) (52).

#### 2.3.4 Ferroptosis

Ferroptosis is a newly recognized form of cell death induced by intracellular iron overload, leading to the accumulation of lipid ROS in the cell membranes. It is closely related to the occurrence of developed inflammatory diseases and is characterized by iron overload and increased ROS levels (53). The membrane lipid repair enzyme glutathione peroxidase 4 (GPX4) is an antioxidant enzyme crucial for protecting cell membranes from oxidation by using glutathione as a cofactor. GPX4 neutralizes lipid peroxides, maintaining membrane fluidity (54). In cases where GPX4 is overexpressed, it can reduce ROS and inhibit ferroptosis (55). Dysfunction of ferroportin 1 (FPN1), an iron-excretory protein, leads to excessive intracellular iron, resulting in ROS production and ultimately contributing to ferroptosis. Knocking out GPX4 in the kidneys of mice has been shown to cause acute renal failure, which can be improved by the use of iron death inhibitors like Liproxstatin-1 (56). The nuclear factor erythroid 2 related factor (NRF2) plays a crucial role in lipid peroxidation and ferroptosis regulation and is an important downstream regulatory target of GPX4 (57). In an *in vitro* model of chicken cardiomyocyte injury induced by uric acid, the NRF2 signaling was inhibited, accompanied by increased lipid accumulation in macrophages and ferroptosis (58). Morphological changes in ferroptosis in renal tubular epithelial cells, such as smaller mitochondria and increased membrane density, have been observed through TEM. Mitochondria are closely associated with ferroptosis (59), and AMPK regulation is linked to mitochondrial homeostasis. Animal experiments have demonstrated that AMPK can inhibit ferroptosis in mice treated with AICAR (an AMPK activator), which has demonstrated a protective effect on kidneys (Figure 3) (60).

In summary, renal inflammation, oxidative stress, and programmed cell death often coexist during the development of GN kidney injury. These processes are interconnected, influencing one another and contributing to the disease’s progression. Uric acid and MSU crystals affect kidney cell morphology and function through the activation of NLRP3 inflammasome and the secretion of related inflammatory factors. This, in turn, results in intracellular organelle dysfunction and contributes to the onset and progression of GN. Understanding the pathogenesis of GN and investigating its causes are valuable for modeling and providing strong evidence for disease diagnosis and treatment. Hyperuricemia, characterized by elevated serum uric acid, is a significant factor in kidney injury, and these elevated uric acid levels play a predominant role in model establishment. The following section summarizes the methods used to establish animal and cellular models of GN, serving as a reference for future model development.

## 3 *In vitro* and *in vivo* models of GN

Avian and mammalian animals are commonly used for GN modeling due to their susceptibility to renal function damage. However, physiological and biochemical differences exist between these animals and humans, limiting their suitability as models. Mammal models provide genetic homogeneity, minor individual variations, and high reproducibility, given their high physiological and biochemical similarities to humans. However, there are some differences in uric acid metabolism. Gouty nephropathy primarily affects higher primates, including humans, due to the lack of a functional urate oxidase (Uox) enzyme. Uox normally metabolizes uric acid into the more water-soluble allantoin, allowing for its easy excretion from the body. However, in primates who lack Uox activity, uric acid levels gradually increase. This excess uric acid forms crystals that can deposit in the kidneys, leading to the formation of uric acid stones over time. These stones can further impair kidney function and contribute to the development of gouty nephropathy. In most mammals, uric acid from purine metabolism is further degraded by the action of Uox to produce the more soluble allantoin to excrete uric acid out of the body, thus making it difficult to form uric acid stones in the kidneys, which rarely produce spontaneous gouty nephropathy. Non-human primates, owing to their evolutionary proximity to humans and physiological similarities, can serve as preferred models when exploring the etiology and pathogenesis of gout.

### 3.1 Avian animal models

Modified diets are commonly employed to induce GN models in avian animals. Hong et al. (61) used 24-week-old male white Henlai chickens and fed them diets with varying protein proportions. After 10 weeks, the high-dose group exhibited significantly higher urate levels, joint deformities, synovial fluid crystal formation, and varying degrees of kidney damage. Importantly, the high-protein diet did not induce adverse side effects, making it suitable for modeling gouty nephropathy. Other methods involve inducing high uric acid phenotypes with renal function damage. This includes elevating uric acid concentration through cisplatin injections in male broilers for 48 h resulting in kidney damage. However, it has been previously reported that uric acid levels in avian blood are commonly used as indicators of kidney function (62). To investigate the impact of a high-purine diet on uric acid levels in birds, 3-week-old broilers were fed a diet supplemented with inosine for 3 weeks, resulting in elevated uric acid concentrations and smaller kidney phenotypes than the normal group (63). Bian et al. (64) conducted a study in which Difak strain quail was raised on a diet containing yeast extract powder. This study revealed a significant increase in uric acid levels from 10 to day 60, along with congestion in renal tissue and interstitial neutrophil infiltration. In another study, Longcheng quail were subjected to a diet enriched with 14% lard stearin and 1% cholesterol in addition to their regular diet. After 14 days, these quail exhibited clinical symptoms, including elevated serum levels, total serum cholesterol, LDL cholesterol, abdominal fat index, and increased xanthine oxidase activity (65). This modeling approach is both stable and easy to use, and it suggests that changes in uric acid and lipid metabolizing enzyme activities may contribute to the pathology of hyperuricemia. Wu et al. (66) conducted a study involving 4-week-old male French quail that were fed a diet containing yeast extract powder and drinking water supplemented with 10% fructose for 30 days. This intervention resulted in noticeable ankle swelling, significantly elevated uric acid levels, synovial fibrous tissue inflammation, massive inflammatory cell infiltration, surrounding tissue edema, and inflammatory kidney damage with the formation of uric acid stones. Poffers et al. (67) observed elevated plasma uric acid concentrations in red-tailed hawks following a high-purine diet. However, it’s worth noting that breeding birds on a large scale can be challenging and costly, limiting the establishment of gouty nephropathy models.

### 3.2 Mammalian models

Mammals continue are the most commonly used animals for establishing models of metabolic diseases. The combination of potassium oxalate, a uricase inhibitor, and incorporation of yeast extract to the diet is commonly employed to increase serum urate concentrations in mice. An increase in serum uric acid and Superoxide dismutase (SOD) values was observed in mice intraperitoneally administered with potassium oxybate (300 mg/kg) for eight consecutive days, suggesting that sustained elevated uric acid can cause oxidative stress (68). Qin et al. (69) established a GN model in rats by administering adenine by gavage. At 21 days, uric acid, urea nitrogen, and creatinine concentrations increased, and renal tubular detachment and necrosis were observed in the kidneys. In the study by Guan et al. (70), selected male C57BL/6J mice to establish a model of gouty nephropathy by gavage of adenine + potassium oxalate (100 + 500 mg/kg) for 3 weeks. The key manifestation was increased serum uric acid level and significant renal damage. Due to the increased intake of adenine, uricase activity was inhibited, leading to gouty nephropathy. NLRP3 gene deficiency can inhibit the development of renal inflammation and fibrosis. Researchers have shown elevated uric acid levels, renal fibrosis, and interstitial leukocyte infiltration in wild-type mice fed with a normal diet, a Western diet (43% fat + 0.15% cholesterol) and 15% fructose with male NLRP3 knockout mice compared with wild-type mice. In contrast, the aforementioned effects were attenuated in NLRP3 knockout mice. This indicated that NLRP3 is a critical regulator in hyperuric acid nephropathy (71). Khattri et al. (72) administered C57BL/6J mice with 0.15% adenine dietary supplement for 6 months. Application of renal metabolomics revealed significantly reduced ATP and DNA levels due to energy stress and impaired mitochondria with renal tubular dysfunction. In Lu et al. (73) employed transcription activator-like effector nuclease (TALEN) technology to develop Uox knockout mouse models in a pure C57BL/6J background (73). These mice exhibited renal insufficiency characterized by both glomerular and tubular lesions. Notably, these models exhibited a relatively high survival rate and mirrored aspects of the uric acid metabolic process seen in humans. However, it’s worth considering more efficient and less cumbersome techniques for model development due to their complexity and associated higher mortality rates. Previously, a model of uric acid nephropathy was established by intraperitoneal administration of uric acid to rats for 4 weeks. Results indicated that elevated serum uric acid levels were relatively stable and that there were fatty oxidase and fibronectin deposits in the kidney which participated in the development of high uric acid-induced kidney injury (74). Elsewhere, 12 adult healthy albino rabbits exhibited significantly elevated uric acid levels after six consecutive weeks of oral administration of fructose syrup following exposure to elevated uric acid, which increased the risk of developing gouty nephropathy (75). Researchers developed a model of hyperuricemia by injecting uric acid into the jugular vein of Swedish long white pigs while adding grains, high fructose, and inosine to their diets. It was found that plasma creatinine concentrations were increased, uric acid excretion was decreased, and renal failure occurred in pigs after 5/6 nephrectomy (76). Studies have shown that Dalmatian dogs exhibit a phenotype in their uric acid metabolism process similar to that of humans. This similarity leads to the spontaneous development of gout in Dalmatians, underscoring the importance of SLC2A9 in uric acid transport (77).

### 3.3 Other animal models

The tree shrew is a close relative of primates and is closer to primates compared to rodents in terms of genetic inheritance. In addition, the tree shrew has a small size, rapid reproduction, easy feeding, among other factors. It is considered the preferred choice for studying diseases (78). Intraperitoneal injection of 80 mg/kg of potassium oxy zincate suspension in tree shrews and blood samples collected via the tail vein after administration showed elevated levels of uric acid and xanthine oxidase (XOD) expression in the liver (79), and the model was stable and reproducible. In recent years, there has been a growing utilization of zebrafish models. Notably, these models offer a high degree of conservation in functional domains when compared to human proteins and exhibit kidney anatomy that is comparable to that of mammals (80). Wei et al. (81) utilized the uricase inhibitor potassium oxybate to establish a model of hyperuricemia in zebrafish. After 24 h, elevated levels of serum uric acid, urea nitrogen, creatinine, and xanthine oxidase activity were detected. The types of animal models and their induction methods are presented in Table 1.

**TABLE 1:** Animal models of gouty nephropathy.

Animals species	Name	Species	Modeling method	Disease cycle	References
Poultry	White Henleys	Birds, Pheasant	High protein diet, 19.11%, p.o	70 days	(61)
	Proto chickens	Birds, Pheasant	Cisplatin, 3.5 mg/mg, i.p	48 h	(62)
	Broiler	Birds, Pheasant	Inosine food, 6.7 mol/kg, p.o	21 days	(63)
	Difacia strain quail	Birds, Pheasant	Yeast extract powder, p.o	60 days	(64)
	Longcheng quail	Birds, Pheasant	Lard stearin + cholesterol, 14 + 1%, p.o	14 days	(65)
	French quail	Birds, Pheasant	Yeast extract + fructose, 10%, p.o	30 days	(66)
	Red-tailed hawk	Ornithischia, Falconiformes, subfamily Buzzard	High purine food, p.o	72 h	(67)
Mammals	Mice	Mammalia, Rodentia Muridae	Oxybate + yeast, 300 mg/kg, i.p	8 days	(68)
	Rats	Mammalia, Rodentia Muridae	Adenine, 300 mg/kg, p.o	21 days	(69)
	Mice	Mammalia, Rodentia Muridae	Adenine +potassium oxybate, 100 + 500 mg/kg	21 days	(70)
	Mice	Mammalia, Rodentia Muridae	Fructose + fat + cholesterol, 0.15 + 43% + 15, p.o	16 weeks	(71)
	Mice	Mammalia, Rodentia Muridae	Adenine, 0.15%, p.o	24 weeks	(72)
	Mice	Mammalia, Rodentia Muridae	Rodentia Knockout uric acid oxidase gene	–	(73)
	Rats	Mammalia, Rodentia Muridae	Uric acid, 250 mg/kg, i.p	28 days	(74)
	Albino rabbits	Mammalia, Rutaceae Fructose	Fructose, 1 g/kg, p.o	42 days	(75)
	Swedish long white pig	Mammalia, Diptera, Suidae	Uric acid + inosine + fructose, 40 mg/ml, i.p + 20 + 0.9%, p.o	14 days	(76)
	Dalmatian	Mammalia, Carnivora Canidae	SLC2A9 gene sequence mutation	–	(77)
Other classes	Tree shrews Zebrafish	Mammalia, Primate family Tree Shrew Scleractinian, Cyprinidae	Oxonate suspension, 80 mg/kg, i.p Potassium oxybate, 125 μg/ml	12, 24 h	(79, 81)

### 3.4 *In vitro* cellular model

Besides animal models, establishing cellular models to simulate human uric acid production and metabolism need to be considered. HK-2 cells are derived from human renal tubular epithelial cells, terminal cells for uric acid production and excretion. Hou et al. (82) stimulated HK-2 cells with adenosine and Xanthine oxidase (XO) as inducers and found that elevated uric acid and hypoxanthine levels. In contrast to animal models, this cellular model boasts a rapid turnover rate, a heightened sensitivity to drug mechanisms, and is better suited for high-throughput screening. Given its numerous advantages, the cellular model can emerge as the top preference for the initial screening of potential natural compounds. As the most commonly used human monocyte, THP-1 is applied in studies of crystal-induced inflammation (83). Xue et al. (84) used MSU crystals to stimulate human myeloid leukemia mononuclear cells (THP-1) cells to establish a gouty inflammatory cell model and observed a significant increase in inflammatory concentration after cell stimulation with MSU. Incorporation of uric acid precursors to the cell culture medium is also an effective approach to induce uric acid production. Adachi et al. (85) screened AML12 mouse hepatocytes for drugs with anti-hyperuricemia activity by culturing them. They opted for uric acid precursor substances, guanosine and inosine, at a concentration of 100 μmol/L each to stimulate hepatocytes. This stimulation enhanced uric acid production in AML12 cells. The isolated cells are reliably reproducible, and the drug doses used are much smaller and suitable for large-scale screening systems (6). MSU crystal deposits are often observed in kidney of GN patients, and MSU crystals can induce inflammatory response. To investigate the effects of MSU on human renal mesangial cells (HRMCs) and intercellular adhesion molecule (ICAM-1) expression, Luo et al. (6) showed that MSU stimulation promoted ICAM-1 adhesion molecule expression and which reached the peak after 8 h. In addition, MSU-induced monocyte expression and intercellular adhesion by regulating TLRs activation of inflammatory factors involved in the pathogenesis of GN. To investigate the relationship between uric acid and renal tubular injury, researchers stimulated rat proximal tubular epithelial cells (NRK-52E) with uric acid. It was observed that uric acid activation in NF-K B enhanced cell proliferation and upregulated levels of uric acid transporter URAT1 and increased fibronectin expression in renal tubular epithelial cells to promote renal fibrosis in a time- and dose-dependent manner (74). In addition, a concentration of 0.1 mmol/L soluble uric acid induced the upregulation α- Smooth muscle actin α-SMA and fibronectin (FN) in rat mesangial cells (HBZY-1) thereby promoting the occurrence of renal fibrosis (86). The types of cell models and their construction methods are summarized in Table 2.

**TABLE 2:** *In vitro* cell models.

Cell selection	Inducer	Construction method	References
HK-2 cells (human renal cortical proximal tubule epithelial cells)	Adenosine and xanthine	Containing 0.5 mmol/L adenosine added in RPMI 1640 medium for 24 h	(82)
THP-1 (human monocytes)	MSU	MSU THP-1 cells were treated with 100 ng/mL PAM for 48 h and then stimulated with 250 μg/mL MSU crystals for 6 h	(83)
AML12 (mouse hepatocytes)	Guanosine and Inosine	containing 100 μM guanosine and inosine, respectively, were stimulated for 2 h	(85)
HRMCs (human renal mesangial cells)	MSU	MSU Cells were stimulated with three doses of MSU at 0.5, 5, and 50 ng/ml and peaked after 8 h	(6)
NRK-52E (rat proximal renal tubular epithelial cells)	UA	NPK-52 cells were inoculated in medium containing 10% fetal bovine serum and then incubated with serum-free medium for 16 h before adding different concentrations of uric acid to stimulate	(74)
HBZY-1 (Rat glomerular thylakoid cells)	UA	Stimulated cells were measured with 0.2 mmol/L concentration of UA after different times of 24, 36, and 48 h	(86)

## 4 Summary and outlook

Considering the importance of hyperuricemia in kidney damage, strategies that decrease uric acid levels may be effective treatments for gouty nephropathy. This study describes the impact of immune system dysfunction and programmed cell death in the regulation of gouty nephropathy. The data reviewed here show that soluble urate and MSU can activate the innate immune system with Nod-like receptor 3 and nuclear factors to promote the release of IL-Iβ inflammatory factors, causing inflammatory responses. They also enhance the activation of NLRP3 inflammasome to trigger pyroptosis and autophagy, and induce sustained increase in uric acid levels leading to renal disease.

Animal experiments serve as crucial tools for exploring disease mechanisms and evaluating drug efficacy. However, establishing stable animal models is a fundamental prerequisite for the success of these experiments. The establishment of animal models provides a reference for exploring the potential mechanisms of hyperuricemia and GN, and the most commonly used experimental animals are mammals and avians which have similar uric acid metabolism pathways as humans. Currently employed modeling techniques predominantly rely on a combined modeling approach, involving the administration of exogenous sodium urate and a high-purine diet as modeling agents. In contrast, endogenously induced gouty stone deposition models are infrequently utilized. These models are characterized by elevated uric acid levels and resultant renal injury, with the primary manifestations being increased uric acid levels and kidney damage. Besides accurately replicating the disease’s pathogenesis, it is imperative to consider the overall health status of the animal, the potential for irreversible damage caused by the modeling agent, as well as the model’s stability and the duration of its cycle when constructing the model. This multifaceted approach ensures the successful establishment of the model. *In vitro* cell models are closer to the actual human disease owing to their advantages such as short research cycle and simple operation which makes them ideal for rapid screening of drugs for the treatment of gouty nephropathy, promoting the validation of conclusions from animal model-based studies. Programmed cell death is contributes to the pathogenesis of GN, but the current studies do not take these factors into consideration during establishment of animal or cellular models. Therefore, new animal and cellular models should be developed to improve drugs discovery and understanding of disease mechanisms.

Selecting the ideal animal model for GN presents a challenge. While birds and mammals offer advantages, significant obstacles exist. Germline differences limit their suitability, and replicating human GN poses difficulties despite some models achieving stable, elevated uric acid levels. Furthermore, their limited lifespans hinder chronic disease modeling. Human evolution led to uricase loss, causing elevated uric acid and kidney injury. While knocking out the uricase gene in model animals can achieve this, the low survival rate, complex technology, high cost, and early stage of development limit its application. Mammals present operational ease, but their divergent uric acid metabolism makes maintaining stable, chronic hyperuricemia difficult. Poultry models offer closer uric acid metabolism pathways to humans and some clinical symptom reflection, but significant limitations arise from genomic differences and specialized feeding requirements. In comparison, non-human primates demonstrate superior surface, construct, and predictive validity for GN (87). Although literature lacks established models, their closer metabolic and genomic relationship to humans holds promise for drug development. Additionally, their longer lifespans and ability to spontaneously develop GN with age offer valuable insights into GN’s causal mechanisms, surpassing the single-environmental-factor conditions seen in other models. In conclusion, while existing models offer insights, non-human primates hold the greatest potential for advancing our understanding and treatment of GN due to their closer resemblance to humans. Therefore, the development and search for potential non-human primate disease models need to be prioritized to improve gouty nephropathy screening and analysis of new drugs. If attainable, this endeavor will not only enhance our comprehension of the underlying mechanisms of GN but will also expedite the early exploration of preventive strategies, as well as the screening and assessment of novel medications aimed at diminishing the risk of morbidity.

## Author contributions

LW: Writing – original draft. XZ: Writing – original draft. JS: Writing – original draft. YW: Writing – review and editing. TZ: Writing – review and editing. NX: Writing – review and editing. XL: Supervision, Writing – review and editing. DQ: Supervision, Writing – review and editing. YX: Writing – review and editing. YZ: Writing – review and editing. JX: Supervision, Writing – review and editing. ZL: Supervision, Writing – review and editing. ZX: Supervision, Writing – review and editing.
